# Availability of access, watch, and reserve (AWaRe) group of antibiotics in community pharmacies located close to a tertiary care hospital in Lalitpur, Nepal

**DOI:** 10.1371/journal.pone.0294644

**Published:** 2023-11-20

**Authors:** Nisha Jha, Bibechan Thapa, Samyam Bickram Pathak, Sajala Kafle, Anish Mudvari, Pathiyil Ravi Shankar

**Affiliations:** 1 Department of Clinical Pharmacology and Therapeutics, KIST Medical College, Lalitpur, Nepal; 2 Department of Emergency Medicine, Kirtipur Hospital, Kirtipur, Nepal; 3 Department of Intensive Care Unit and Critical care, Nepal Mediciti Hospital, Sainbu, Bhaisepati, Nepal; 4 Department of Pharmacology, Maharajgunj Medical Campus, Maharajgunj, Kathmandu, Nepal; 5 IMU Centre for Education, International Medical University, Kuala Lumpur, Malaysia; North Carolina State University, UNITED STATES

## Abstract

**Introduction:**

The access, watch, and reserve (AWaRe) classification of antibiotics was developed in 2019 by the WHO Expert Committee on the Selection and Use of Essential Medicines as a tool to support antibiotic stewardship efforts at local, national, and global levels. The objectives of this study were to assess the availability of antibiotics as per WHO AWaRe classification at community pharmacies located around a tertiary care hospital in Lalitpur and to compare these antibiotics with the national essential medicine list of Nepal.

**Method:**

The cross-sectional study was conducted at community pharmacies located within a two-kilometer radius of a teaching hospital from August to November 2022. A total of 82 community pharmacies registered with the Nepal Chemist and Druggists Association and the Department of Drug Administration were studied. Data was collected using a standard proforma containing the names of the antibiotics classified as per the WHO’s AWaRe classification.

**Results:**

Access group of antibiotics, Ampicillin, (82;100%), Amoxycillin, (82;100%), Flucloxacillin, (82;100%), and Metronidazole, (82;100%) were available in all community pharmacies. Results from the watch group showed that Azithromycin, (80; 97.6%) was available in all pharmacies followed by Cefixime, (80; 97.6%), Ciprofloxacin, (73; 89%), Levofloxacin, (74; 90.2%)and Ofloxacin, (74; 90.2%). Linezolid, (24; 29.3%) was the most common antibiotics available from the reserve group of antibiotics. Colistin was the second commonly available antibiotic. The most available antibiotic from the not recommended group were Ampicillin/Cloxacillin (82; 100%), followed by Piperacillin/Sulbactam, (39; 47.6%). There were differences in the classification of antibiotics between the WHO AWaRe list and the Essential Medicines list of Nepal in terms of numbers of antibiotics listed.

**Conclusion:**

Antibiotics from the not recommended and reserve groups were commonly available in community pharmacies. The implementation of antibiotic guidelines should be emphasized along with strict monitoring of the sale of antibiotics without a prescription in community pharmacy settings.

## Introduction

Antimicrobial resistance (AMR) is a pressing global problem and threatens the very core of modern medicine. Common infections are difficult to treat due to the emergence of drug resistance bacteria [1]. An estimated 0.7 million people may die annually due to the emergence of drug resistance, and this could lead to 10 million deaths by 2050 if no actions are considered for mitigation of the problem [2]. If AMR is not addressed promptly then a post-antibiotic era is inevitable where even common infections will pose higher mortality [3].

Nepal is a low-and-middle-income country (LMICs) with a high burden of infectious diseases and antimicrobial resistance related to human and economic losses [4–6]. Many factors lead to the emergence of antimicrobial resistance like self-medication, availability of antibiotics as an over-the-counter drug, taking an incomplete course of antibiotics and lack of proper regulation [7].

The overall use and consumption of antibiotics has increased by 65% worldwide and this increase is majorly contributed to by the LMICs [8]. Data shows antimicrobial consumption has increased from 3.2 to 6.5 billion daily defined doses (DDD) [8]. Another study also highlighted the maximum use of broad-spectrum antibiotics in India [9, 10]. Data from Nepal is still being collected.

Antibiotics have been classified into Access, Watch, and Reserve (AWaRe) by the World Health Organization (WHO) in 2019. The aim is to restrict the use of antibiotics belonging to watch and reserve groups [11]. This aim of the classification mentions the access group as one acting on a wider range of causative organisms with a lower potential of developing antimicrobial resistance than the drugs from the other two groups. Some examples of this group are benzathine penicillin, amoxicillin, ampicillin, trimethoprim and sulfamethoxazole. Another is the ‘Watch’ group, which has drugs with a higher resistance developing potential and includes antibiotics such as third generation cephalosporins, fluroquinolones and carbapenems. The last group is the ‘Reserve’ group and includes the antibiotics reserved for treating confirmed and suspected cases of multi drug resistant organisms [11]. There are also some antibiotics which are grouped as not recommended including the fixed dose combinations. Commonly, combinations of broad-spectrum antibiotics are not recommended for use in clinical practice [11].

In Nepal, pharmacies are regulated by the drug regulatory authority, Department of Drug Administration as per the Drugs Act 2035 BS. Clauses 17 and 27 of the Drug Act mention that antibiotics should only be prescribed by medical doctors and dispensed by a pharmacy professional [12]. There is an updated national action plan for the use of antibiotics, but the final endorsement for the implementation from the government is not yet complete [13].

Pharmacy professionals working in community settings are important professionals to promote the rational use of medicines in communities. They are commonly seen as the first contact point for seeking healthcare in the community [7]. The common availability of antibiotics as an over-the-counter medicine also encourages self-medication and misuse of antibiotics in Nepal, like in many other low- and middle-income countries (LMICs) [7].

The objectives of this study were to assess the availability of antibiotics as per the WHO AWaRe classification at community pharmacies located within a two-kilometer radius of a tertiary care hospital in Lalitpur and to compare these with the national essential medicine list of Nepal.

## Methodology

### Study design

The cross-sectional study was conducted at community pharmacies located within a two-kilometer radius of a particular teaching hospital.

### Study population

Community pharmacies located within a two-kilometer radius of the teaching hospital. Google Maps was used to identify pharmacies located within two kilometers from the hospital premises. A total of 82 community pharmacies registered with the Nepal Chemist and Druggists Association and the Department of Drug Administration were studied. The study was conducted from August to November 2021.

In Nepal, pharmacies are often clustered around hospitals even if the hospital has its own pharmacy. We selected community pharmacies within a two-kilometer (driving distance) of the KIST Medical College Teaching Hospital to study the availability of AWaRe group of antibiotics. This radius includes densely populated areas of Lalitpur sub-metropolitan city and Mahalaxmi municipality. At the time of the study there were 82 pharmacies within the 2 km radius of the KIST Medical College Teaching Hospital according to data from the Bulletin published by the Department of Drug Administration (DDA) [14] and based on resources available, 82 (15%) pharmacies were approached, and all consented to participate in the study.

### Data collection tool and methods

Data was collected using a self-administered standard proforma containing the names of the antibiotics classified as per the WHO’s AWaRe classification. The tool contained different sections for the location and name of the community pharmacies, the educational qualification of the pharmacy professionals and their working experiences. Other sections contained names of the antibiotics as per access, watch, reserve, and not recommended groups. Not recommended group of antibiotics are fixed dose combinations having multiple broad-spectrum antibiotics.

A data collector with a pharmacy background was hired and trained in the process of data collection. The questionnaire was explained to the data collector who was also trained in the process of obtaining written informed consent prior to data collection.

The data collection tool was developed based on the names of the antibiotics. The names of antibiotics from each category were taken from the essential medicine list published by WHO in 2019 [11]. The data collection tool is shown as a S1 File.

The content and face validity were ensured by sending the questionnaire to three content experts for their comments and suggestions. Pretesting was done among five community pharmacies not located within the two-kilometer radius of KIST Medical College Teaching Hospital and this data was not included in the main data. The presence of a particular antibiotic in a particular pharmacy was noted across different categories. If the antibiotic was present a score of 1 was given and if it was absent the score given was 0. The total score for each pharmacy across different groups of antibiotics was noted. The distribution of these scores was compared using a one-sample K-S test. The scores were not normally distributed, and hence median and interquartile range were used to describe the data.

#### Ethical approval

The study was ethically approved by the Institutional Review Board of the Medical College with an approval number of 2078/79/06.

## Results

More than half, (51; 62.1%) of the participants working in the pharmacies were aged between 20–30 years, with a majority of males (47; 57.7%). The maximum number of participants, (45; 54.9%) were having diploma in pharmacy and the working experience was one to five years for the majority, (57; 69.5%) of the participants from the community pharmacies as shown in Table 1.

**Table 1:** Demographics of the pharmacists working in the selected community pharmacies (Lalitpur, 2021).

Variables	Number (Percentage)
**Age (years)**	
20–30	51 (62.1)
31–40	31 (37.9)
**Gender**	
Male	47 (57.7)
Female	35 (42.3)
**Educational Qualification**	
Diploma in Pharmacy	45 (54.9)
Bachelor in Pharmacy	18 (22)
Certified Medical Assistants (CMA)	7 (8.5)
Nurse	12 (14.6)
**Working experience**	
Less than one year	7 (8.5)
One to five years	57 (69.5)
More than five years	18 (22)

Antibiotics like Ampicillin, Amoxycillin, Flucloxacillin, and Metronidazole were available across all the community pharmacies from the access group. Clindamycin, 81 (98.8%) and Amoxycillin and Clavulanic acid, 79 (96.3%) and Oxacillin, 74 (90.2%) were also available from this group as shown in Table 2.

**Table 2:** Availability of antibiotics as per AWaRe groups (Lalitpur, 2021).

SN	Name of the antibiotic (Access Group)	Number (Percentage)	Present in national Essential Medicine List
1	Amikacin	66 (80.5)	Yes
2	Amoxicillin	82 (100)	Yes
3	Amoxycillin Clavulanic Acid	79 (96.3)	No
4	Ampicillin	82 (100)	Yes
5	Chloramphenicol	40 (48.8)	Yes
6	Clindamycin	81 (98.8)	No
7	Cloxacillin	46 (56.1)	Yes
8	Doxycycline	41 (50)	Yes
90	Flucloxacillin	82 (100)	No
10	Gentamicin	17 (20.7)	Yes
11	Metronidazole Oral	82 (100)	Yes
12	Metronidazole IV	20 (24.4)	Yes
13	Nitrofurantoin	82 (100)	Yes
14	Oxacillin	74 (90.2)	No
15	Tetracycline	16 (19.5)	No
	**Watch Group**		
16	Azithromycin	82 (100)	Yes
17	Cefixime	80 (97.6)	Yes
18	Ceftriaxone	13 (15.9)	No
19	Ciprofloxacin	73 (89)	Yes
20	Clarithromycin	14 (17.1)	Yes
21	Erythromycin	10 (12.2)	Yes
22	Fusidic Acid	72 (87.8)	No
23	Gatifloxacin	72 (87.8)	No
24	Levofloxacin	74 (90.2)	No
25	Neomycin	35 (42.7)	No
26	Ofloxacin	74 (90.2)	No
27	Tobramycin	64 (78)	No
28	Vancomycin Oral	3 (3.7)	No
29	Vancomycin IV	6 (7.3)	No
	**Reserve Group**		
30	Colistin	3 (3.7)	Yes, But in the complementary list
31	Linezolid	24 (29.3)	Yes, But in the complementary list
	**Not Recommended Group**		
32	Ampicillin and Cloxacillin	82 (100)	No
33	Ceftriaxone and Tazobactam	28 (34.1)	No
34	Cefuroxime axetil and Linezolid	19 (23.2)	No
35	Meropenam and Sulbactam	18 (22)	No
36	Piperacillin and Sulbactam	39 (47.6)	No

The essential medicine list (EML) of Nepal has some differences in the list of antibiotics as compared to the WHO list for AWaRe antibiotics [15]. The EML of Nepal includes only 15 antibiotics in the access group and one antibiotic in complementary list. Cefazoline has been listed in the complementary list of access groups.

WHO essential medicine list has 42 antibiotics. The names of antibiotics in the WHO list have been listed in the questionnaire. Similarly, the EML of Nepal has 7 antibiotics in the watch group and 4 antibiotics in the complementary list, whereas the WHO list contains 109 antibiotics listed in the questionnaire.

Results from the watch group showed that Azithromycin, (82;100%) was available in all the pharmacies followed by Cefixime (80;97.6%), Ciprofloxacin (73;89%), Levofloxacin (74; 90.2%) and Ofloxacin (74;90.2%). Similarly, Gatifloxacin (72;87.8%), Fusidic acid (72; 87.8%) were also found in the community pharmacies as shown in Table 2.

Linezolid (24;29.3%) was the most common antibiotic available from the reserve group of antibiotics as shown in Table 2. The EML of Nepal has four antibiotics from the reserve group and 2 antibiotics in the complementary list, whereas in the WHO list, the number of antibiotics is 21 as shown in the questionnaire.

The most commonly available antibiotic from the not recommended group was Ampicillin/ Cloxacillin (82;100%). followed by Piperacillin/Sulbactam, (39;47.6%) as shown in Table 1. The essential medicine list of Nepal has no information about the not recommended group of antibiotics, but the WHO list has about 103 names in the not recommended group. Among these names, the study findings showed the presence of 5 combination antibiotics as shown in Table 1.

**Fig 1:**
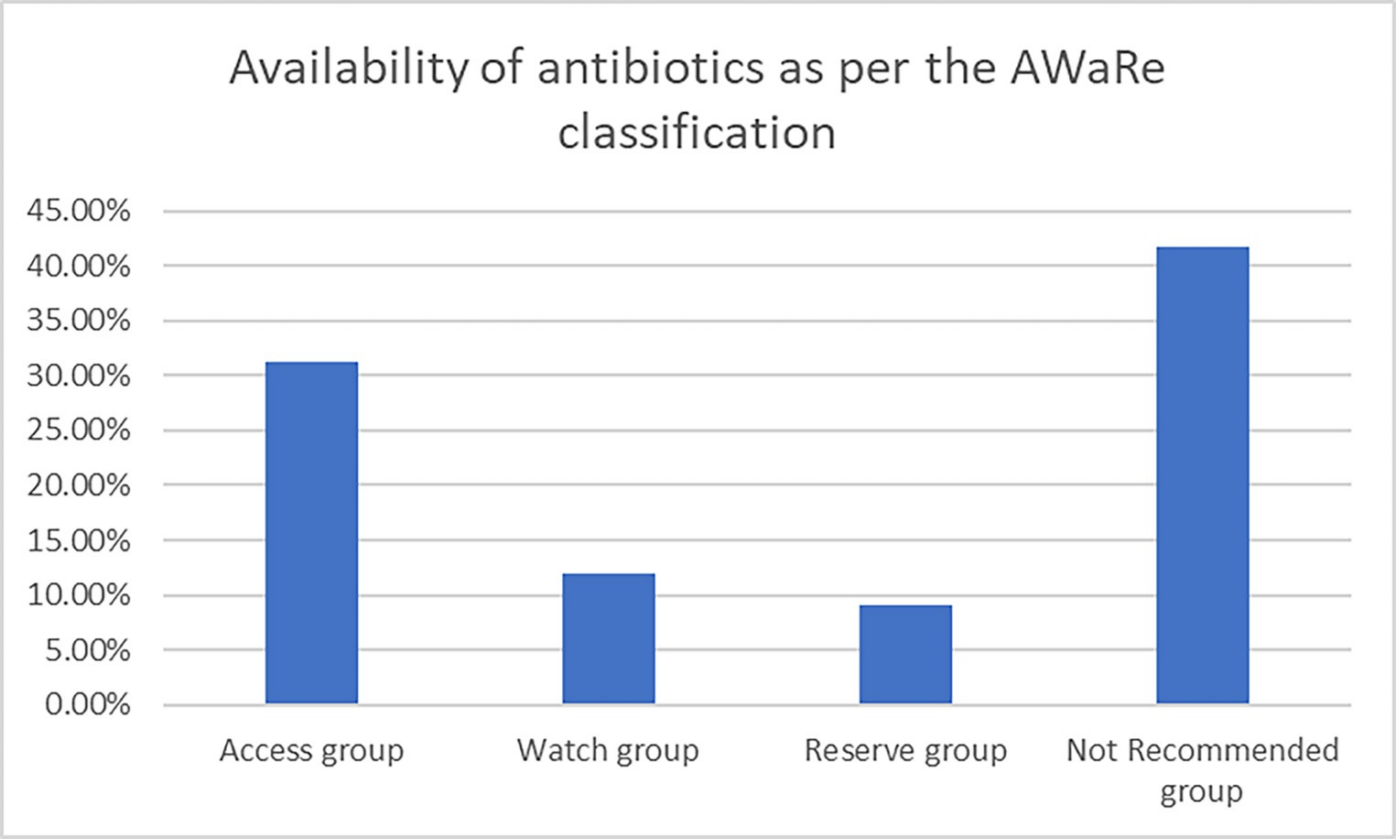
Percentage availability of antibiotics from AWaRe criteria.

The availability of the antibiotics was maximal from the access group, followed by watch and reserve groups as shown in Fig 1.

Fig 1 shows the percentage availability of all the groups of antibiotics. Access group of antibiotics was maximally available followed by the watch group. Similarly, the reserve and not recommended groups were also available in the community pharmacies.

## Discussion

The WHO has classified antibiotics into three major categories as Access, Watch, and Reserve (AWaRe) to support antimicrobial stewardship efforts at local, regional, and global levels. Additionally, they have also added the not recommended group of antibiotics.

In Nepal, antibiotics are not classified as access, watch, and reserve in the national essential medicine list (EML). The sixth revision of the EML of Nepal contains 398 medicines. The list was revised based on the disease prevalence, relevance to public health, safety evidence, the effectiveness of the medicines along with the cost-effectiveness for addressing basic healthcare needs in 2021 [15]. Nepal’s Essential Medicine List has a Complementary List having a (c) symbol placed next to the name of the medicine and denotes medicine(s) that require specialist, diagnostic or monitoring facilities, and/or specialist medical care, and/or specialist training for their use in children. Medicines may also be listed as Complementary based on consistently higher costs or less attractive cost-effectiveness in a variety of settings.

The 2021 update of the AWaRe classification includes an additional 78 antibiotics not previously classified, bringing the total to 258 [16]. The classification is useful for monitoring antibiotic consumption, defining targets, and studying the effects of stewardship policies that aim to optimize antibiotic use and curb antimicrobial resistance. The access group includes 87 antibiotics with activity against a wide range of commonly encountered susceptible pathogens while also showing lower resistance potential than antibiotics in the other groups [16]. Empiric first or second-choice treatment options for common infections are included [14].

Watch group of antibiotics (142 antibiotics) includes antibiotic classes that have higher resistance potential and include most of the highest priority agents among the critically important antimicrobials for human medicine [5] and/or antibiotics that are at relatively high risk of selection of bacterial resistance. These should be prioritized as key targets of local and national stewardship programs and monitoring [3]. The Reserve group (29 antibiotics) includes antibiotics that should be treated as ‘last-resort’ options, or tailored to highly specific patients and settings, and when other alternatives would be inadequate or have already failed [16]. These medicines should be protected and prioritized as key targets of high-intensity national and international stewardship programs involving monitoring and utilization reporting, to preserve their effectiveness [3].

Wider availability of antibiotics in the community pharmacies from the reserve and the not recommended groups can promote the spread of antimicrobial resistance. A recent study done in Nepal has shown dispensing of antibiotics without prescription by 85% of the community pharmacy staff highlighting the irrational dispensing practices for antibiotics. The study also showed the highest dispensing for Amoxicillin followed by Azithromycin [7]. The AWaRe classification is not used in the national essential medicines list and in Nepal many community pharmacies are also located around hospitals and nursing homes. This has been observed by the authors and their main clientele are the hospital inpatients and outpatients. The hospital pharmacy service guidelines specify that all hospitals should run their own pharmacy and the details of operation of pharmacy at different hospitals have been clarified [17]. A recent article mentions that while government hospitals run their own pharmacies, most private hospitals have yet to do so and the monitoring and regulatory mechanisms for hospital pharmacies are still in development [18]. Hence many community pharmacies located near teaching hospitals may be functioning as hospital pharmacies and may require the provision of antibiotics from the watch and reserve categories. Though KIST Medical College runs its own hospital pharmacy, community pharmacies around the hospital may also be competing for clientele. The other challenge is the availability of fixed dose combination (FDC) of antibiotics. The rationality of the use of FDC of ampicillin and cloxacillin has been questioned and has been mentioned as an irrational combination requiring intervention [19]. However, the FDC was shown to be widely available in the current study.

A study from Bangladesh has shown that a pharmacist should be present for dispensing antibiotics [20]. In Nepal, the drug act mentions that the sales and distribution of antibiotics should be strictly done by pharmacists and should not be dispensed without a medical doctor’s prescription [8]. A study suggests the need for regulation of the sales and distribution of antibiotics without a doctor’s prescription in community pharmacies [21].

Nepal has framed a National Action Plan (NAP) for AMR in 2014 but this has not been implemented yet [22]. This challenge is like that seen in Bangladesh [20]. According to the Department of Drug Administration (www.dda.gov.np), there are around 28,572 registered allopathic community pharmacies in Nepal. This may have an important impact on the use of antibiotics. Currently, there are 128 pharmaceutical companies registered in Nepal. About 51 domestic pharmaceutical companies produce antibiotics locally and 280 international pharmaceutical companies produce and distribute antibiotics in the Nepalese market [22].

Another Nepalese study has also shown the maximum sales and distribution of Amoxicillin and Azithromycin from the community pharmacies in two districts of the country [21]. The published study data shows that the antibiotics from access and watch groups are more sold and dispensed. There is no data available for antimicrobials consumption to date and the work for this is being conducted by the national drug regulatory authority of Nepal, the Department of Drug Administration in collaboration with the Fleming Fund. The situation may be exacerbated by the COVID-19 pandemic. A recent study has shown the use of cephalosporins and macrolides in COVID treatment [23]. This is very similar to a study published recently in Bangladesh [24].

Reserve group of antibiotics should not be available in community settings and should be restricted to hospital settings. The availability of the reserve group of antibiotics can enhance the irrational use of antibiotics in community settings, exacerbating the spread of antimicrobial resistance in humans. There are no studies published for antibiotic dispensing from community pharmacies as per the WHO’s AWaRe criteria in Nepal. Thus, this study will be providing baseline data for the availability of antibiotics in community settings. This can be seen as a strength of the study. This AWaRe classification has been adopted by the national drug regulatory authority, but the term not recommended group is not used. Antibiotics should not be sold and dispensed without a doctor’s prescription, but due to the lack of stringent regulatory monitoring, this has been practiced in Nepal. The hospital pharmacies do have all the antibiotics available for the patient’s treatment, but there is no recommendation for antibiotics for the pharmacies located near a hospital.

### Limitations

The dosage forms and strengths of the individual antibiotic were not recorded. The availability of antibiotics was only studied at a particular time. A longitudinal study was not done, and hence seasonal and other variations were not explored. This study may not be generalized as only a small number of pharmacies were included in the study.

## Conclusion and recommendation

The most available antibiotics were from access, followed by watch group, reserve, and not recommended groups. Ampicillin was the common antibiotic from the access group and Azithromycin from the watch group. Ampicillin/Cloxacillin was the most common antibiotic from the not recommended group and Linezolid from the reserve group of antibiotics. Antibiotics from the watch, reserve and not recommended group were available in the community pharmacies. The current antibiotic guidelines focus on the use of antibiotics in the treatment of infections. We were not able to find guidelines regarding the antibiotics that should be available in community pharmacies and those that should be restricted. Many community pharmacies located near hospitals and nursing homes may be meeting the requirements of hospital inpatients and outpatients. This makes it difficult to frame guidelines on the availability of antibiotics in community pharmacies.

The literature shows that antibiotics are dispensed without a prescription from community pharmacies in Nepal and the availability of these antibiotics in the pharmacies can led to their widespread use and promote antibiotic resistance. The regulatory authorities may need to regulate the availability of reserve and not recommended antibiotics in community pharmacies in the country to preserve the power of antibiotics. The not recommended FDC of ampicillin and cloxacillin (along with other FDC antibiotics) continues to be widely available and restrictions on its manufacture and sale may need to be considered. The implementation of antibiotic guidelines should be emphasized along with strict monitoring for the sales of antibiotics without a prescription in the community pharmacy settings.

## Supporting information

S1 File (DOCX)
