# Supplementary material for: Availability of access, watch, and reserve (AWaRe) group of antibiotics in community pharmacies located close to a tertiary care hospital in Lalitpur, Nepal
Source: PLoS One. 2023 Nov 20;18(11):e0294644. doi: 10.1371/journal.pone.0294644 (PMC10659150; doi:10.1371/journal.pone.0294644)
Supplement: S1 File — (DOCX) [file pone.0294644.s001.docx]

**Section -1: Information on antibiotics**

**Access Group of antibiotics**

| S.N. | Antibiotics | Category | Yes | No |
| --- | --- | --- | --- | --- |
| 1. | Amikacin | Aminoglycosides |  |  |
| 2. | Amoxicillin | Penicillins |  |  |
| 3. | Amoxicillin/clavulanic Acid | Beta lactam - beta lactamase inhibitor |  |  |
| 4. | Ampicillin | Penicillins |  |  |
| 5. | Ampicillin/sulbactam | Beta lactam - beta lactamase inhibitor |  |  |
| 6. | Bacampicillin | Penicillins |  |  |
| 7. | Benzathine benzylpenicillin | Penicillins |  |  |
| 8. | Benzylpenicillin | Penicillins |  |  |
| 9. | Cefacetrile | First-generation cephalosporins |  |  |
| 10. | Cefadroxil | First-generation cephalosporins |  |  |
| 11. | Cefalexin | First-generation cephalosporins |  |  |
| 12. | Cefalotin | First-generation cephalosporins |  |  |
| 13. | Cefapirin | First-generation cephalosporins |  |  |
| 14. | Cefatrizine | First-generation cephalosporins |  |  |
| 15. | Cefazedone | First-generation cephalosporins |  |  |
| 16. | Cefazolin | First-generation cephalosporins |  |  |
| 17. | Cefradine | First-generation cephalosporins |  |  |
| 18. | Cefroxadine | First-generation cephalosporins |  |  |
| 19. | Ceftezole | First-generation cephalosporins |  |  |
| 20. | Chloramphenicol | Amphenicols |  |  |
| 21. | Clindamycin | Lincosamides |  |  |
| 22. | Clometocillin | Penicillins |  |  |
| 23. | Cloxacillin | Penicillins |  |  |
| 24. | Dicloxacillin | Penicillins |  |  |
| 25. | Doxycycline | Tetracyclines |  |  |
| 26. | Flucloxacillin | Penicillins |  |  |
| 27. | Gentamicin | Aminoglycosides |  |  |
| 28. | Mecillinam | Penicillins |  |  |
| 29. | Metronidazole IV | Imidazoles |  |  |
| 30. | Metronidazole Oral | Imidazoles |  |  |
| 31. | Nafcillin | Penicillins |  |  |
| 32. | Nitrofurantoin | Nitrofurantoin |  |  |
| 33. | Oxacillin | Penicillins |  |  |
| 34. | Penamecillin | Penicillins |  |  |
| 35. | Phenoxymethylpenicillin | Penicillins |  |  |
| 36. | Pivampicillin | Penicillins |  |  |
| 37. | Pivmecillinam | Penicillins |  |  |
| 38. | Procainebenzylpenicillin | Penicillins |  |  |
| 39. | Spectinomycin | Aminocyclitols |  |  |
| 40. | Sulfadiazine/Trimethoprim | Trimethoprim - sulfonamide combinations |  |  |
| 41. | Sulfamethizole/Trimethoprim | Trimethoprim - sulfonamide combinations |  |  |
| 42. | Sulfamethoxazole/Trimethoprim | Trimethoprim - sulfonamide combinations |  |  |
| 43. | Sulfametrole/Trimethoprim | Trimethoprim - sulfonamide combinations |  |  |
| 44. | Sulfamoxole/Trimethoprim | Trimethoprim - sulfonamide combinations |  |  |
| 45. | Sultamicillin | Beta lactam - beta lactamase inhibitor |  |  |
| 46. | Tetracycline | Tetracyclines |  |  |
| 47. | Thiamphenicol | Amphenicols |  |  |
| 48. | Trimethoprim | Trimethoprim |  |  |

**Watch Group of antibiotics**

| S.N. | Antibiotics | Category | Yes | No |
| --- | --- | --- | --- | --- |
| 1. | Arbekacin | Aminoglycosides |  |  |
| 2. | Azithromycin | Macrolides |  |  |
| 3. | Azlocillin | Penicillins |  |  |
| 4. | Biapenem | Carbapenems |  |  |
| 5. | Carbenicillin | Second-generation cephalosporins |  |  |
| 6. | Cefaclor | Second-generation cephalosporins |  |  |
| 7. | Cefamandole | Second-generation cephalosporins |  |  |
| 8. | Cefbuperazone | Third-generation cephalosporins |  |  |
| 9. | Cefcapenepivoxil | Third-generation cephalosporins |  |  |
| 10. | Cefdinir | Third-generation cephalosporins |  |  |
| 11. | Cefepime | Fourth-generation cephalosporins |  |  |
| 12. | Cefetametpivoxil | Third-generation cephalosporins |  |  |
| 13. | Cefixime | Third-generation cephalosporins |  |  |
| 14. | Cefmenoxime | Third-generation cephalosporins |  |  |
| 15. | Cefmetazole | Second-generation cephalosporins |  |  |
| 16. | Cefminox | Second-generation cephalosporins |  |  |
| 17. | Cefodizime | Third-generation cephalosporins |  |  |
| 18. | Cefonicid | Second-generation cephalosporins |  |  |
| 19. | Cefoperazone | Third-generation cephalosporins |  |  |
| 20. | Ceforanide | Second-generation cephalosporins |  |  |
| 21. | Cefoselis | Fourth-generation cephalosporins |  |  |
| 22. | Cefotaxime | Third-generation cephalosporins |  |  |
| 23. | Cefotetan | Second-generation cephalosporins |  |  |
| 24. | Cefotiam | Second-generation cephalosporins |  |  |
| 25. | Cefotiamhexetil | Second-generation cephalosporins |  |  |
| 26. | Cefoxitin | Second-generation cephalosporins |  |  |
| 27. | Cefozopran | Fourth-generation cephalosporins |  |  |
| 28. | Cefpiramide | Third-generation cephalosporins |  |  |
| 29. | Cefpirome | Fourth-generation cephalosporins |  |  |
| 30. | Cefpodoximeproxetil | Third-generation cephalosporins |  |  |
| 31. | Cefprozil | Second-generation cephalosporins |  |  |
| 32. | Ceftazidime | Third-generation cephalosporins |  |  |
| 33. | Cefterampivoxil | Third-generation cephalosporins |  |  |
| 34. | Ceftibuten | Third-generation cephalosporins |  |  |
| 35. | Ceftizoxime | Third-generation cephalosporins |  |  |
| 36. | Ceftriaxone | Third-generation cephalosporins |  |  |
| 37. | Cefuroxime | Second-generation cephalosporins |  |  |
| 38. | Chlortetracycline | Tetracyclines |  |  |
| 39. | Ciprofloxacin | Fluoroquinolones |  |  |
| 40. | Clarithromycin | Macrolides |  |  |
| 41. | Clofoctol | Phenol derivatives |  |  |
| 42. | Delafloxacin | Fluoroquinolones |  |  |
| 43. | Dibekacin | Aminoglycosides |  |  |
| 44. | Dirithromycin | Macrolides |  |  |
| 45. | Doripenem | Carbapenems |  |  |
| 46. | Enoxacin | Fluoroquinolones |  |  |
| 47. | Ertapenem | Carbapenems |  |  |
| 48. | Erythromycin | Macrolides |  |  |
| 49. | Fleroxacin | Fluoroquinolones |  |  |
| 50. | Flomoxef | Second-generation cephalosporins |  |  |
| 51. | Flumequine | Fluoroquinolones |  |  |
| 52. | Fosfomycinoral | Phosphonics |  |  |
| 53. | FusidicAcid | Steroid antibacterials |  |  |
| 54. | Garenoxacin | Fluoroquinolones |  |  |
| 55. | **Garenoxacin** | Fluoroquinolones |  |  |
| 56. | Gatifloxacin | Fluoroquinolones |  |  |
| 57. | Imipenemcilastatin | Carbapenems |  |  |
| 58. | Isepamicin | Aminoglycosides |  |  |
| 59. | Josamycin | Macrolides |  |  |
| 60. | Kanamycin | Aminoglycosides |  |  |
| 61. | Latamoxef | Third-generation cephalosporins |  |  |
| 62. | Levofloxacin | Fluoroquinolones |  |  |
| 63. | Lincomycin | Macrolides |  |  |
| 64. | Lomefloxacin | Fluoroquinolones |  |  |
| 65. | Lymecycline | Tetracyclines |  |  |
| 66. | Meropenem | Carbapenems |  |  |
| 67. | Metacycline | Tetracyclines |  |  |
| 68. | Mezlocillin | Penicillins |  |  |
| 69. | Micronomicin | Aminoglycosides |  |  |
| 70. | Midecamycin | Macrolides |  |  |
| 71. | Minocyclineoral | Tetracyclines |  |  |
| 72. | Moxifloxacin | Fluoroquinolones |  |  |
| 73. | Neomycin | Aminoglycosides |  |  |
| 74. | Netilmicin | Aminoglycosides |  |  |
| 75. | Norfloxacin | Fluoroquinolones |  |  |
| 76. | Ofloxacin | Fluoroquinolones |  |  |
| 77. | Oleandomycin | Macrolides |  |  |
| 78. | Oxytetracycline | Tetracyclines |  |  |
| 79. | Panipenem | Carbapenems |  |  |
| 80. | Pazufloxacin | Fluoroquinolones |  |  |
| 81. | Pefloxacin | Fluoroquinolones |  |  |
| 82. | Pheneticillin | Penicillins |  |  |
| 83 | Piperacillin | Penicillins |  |  |
| 84. | Piperacillintazobactam | Beta lactam - beta lactamase inhibitor (anti-pseudomonal) |  |  |
| 85. | Pristinamycin | Streptogramins |  |  |
| 86. | Prulifloxacin | Fluoroquinolones |  |  |
| 87. | Ribostamycin | Aminoglycosides |  |  |
| 88. | Rifabutin | Rifamycins |  |  |
| 89. | Rifampicin | Rifamycins |  |  |
| 90. | Rifamycin | Rifamycins |  |  |
| 91. | Rifaximin | Rifamycins |  |  |
| 92. | Roxithromycin | Macrolides |  |  |
| 93. | Rufloxacin | Fluoroquinolones |  |  |
| 94. | Sisomicin | Aminoglycosides |  |  |
| 95. | Sitafloxacin | Fluoroquinolones |  |  |
| 96. | Sparfloxacin | Fluoroquinolones |  |  |
| 97. | Spiramycin | Macrolides |  |  |
| 98. | Spiramycin/metronidazole | Combination of antibiotics |  |  |
| 99. | Streptomycin | Aminoglycosides |  |  |
| 100. | Sulbenicillin | Penicillins |  |  |
| 101. | Tebipenem | Carbapenems |  |  |
| 102. | Teicoplanin | Glycopeptides |  |  |
| 103. | Telithromycin | Macrolides |  |  |
| 104. | Temocillin | Carboxypenicillins |  |  |
| 105. | Ticarcillin | Carboxypenicillins |  |  |
| 106. | Tobramycin | Aminoglycosides |  |  |
| 107. | Tosufloxacin | Fluoroquinolones |  |  |
| 108. | VancomycinIV | Glycopeptides |  |  |
| 109. | Vancomycinoral | Glycopeptides |  |  |

**Reserve Group of antibiotics**

| S.N. | Antibiotics | Category | Yes |  |
| --- | --- | --- | --- | --- |
| 1. | Aztreonam | Monobactams |  |  |
| 2. | Ceftaroline fosamil | Fifth-generation cephalosporins |  |  |
| 3. | Ceftazidime-avibactam | Third-generation cephalosporins |  |  |
| 4. | Ceftobiprole medocaril | Fifth-generation cephalosporins |  |  |
| 5. | Ceftolozane-tazobactam | Fifth-generation cephalosporins |  |  |
| 6. | Colistin | Polymyxins |  |  |
| 7. | Dalbavancin | Glycopeptides |  |  |
| 8. | Dalfopristin-quinupristin | Streptogramins |  |  |
| 9. | Daptomycin | Lipopeptides |  |  |
| 10. | Eravacycline | Tetracyclines |  |  |
| 11. | Faropenem | Penems |  |  |
| 12. | Fosfomycin (IV) | Phosphonics |  |  |
| 13. | Linezolid | Oxazolidinones |  |  |
| 14. | Meropenem-vaborbactam | Carbapenems |  |  |
| 15. | Minocycline (IV) | Tetracyclines |  |  |
| 16. | Omadacycline | Tetracyclines |  |  |
| 17. | Oritavancin | Glycopeptides |  |  |
| 18. | Plazomicin | Aminoglycosides |  |  |
| 19. | Polymyxin B | Polymyxins |  |  |
| 20. | Tedizolid | Oxazolidinones |  |  |
| 21. | Telavancin | Glycopeptides |  |  |
| 22. | Tigecycline | Glycylcyclines |  |  |
| **S.N.** | **Not recommended Antibiotics** | | **Yes** | **No** |
| 1. | Acetylspiramycin/metronidazole | |  |  |
| 2. | Amikacin/cefepime | |  |  |
| 3. | Amoxicillin/bacillus coagulans/cloxacillin | |  |  |
| 4. | Amoxicillin/clavulanic acid/lactic ferments | |  |  |
| 5. | Amoxicillin/clavulanic acid/lactobacillus acidophilus | |  |  |
| 6. | Amoxicillin/clavulanic acid/nimesulide | |  |  |
| 7. | Amoxicillin/cloxacillin | |  |  |
| 8. | Amoxicillin/cloxacillin/lactic acid | |  |  |
| 9. | Amoxicillin/cloxacillin/lactobacillus acidophilus/serrapeptase | |  |  |
| 10. | Amoxicillin/cloxacillin/lactobacillus lactis | |  |  |
| 11. | Amoxicillin/cloxacillin/serrapeptase | |  |  |
| 12. | Amoxicillin/dicloxacillin | |  |  |
| 13 | Amoxicillin/dicloxacillin/saccharomyces boulardii | |  |  |
| 14 | Amoxicillin/flucloxacillin | |  |  |
| 15 | Amoxicillin/flucloxacillin/lactobacillus acidophilus | |  |  |
| 16 | Amoxicillin/metronidazole | |  |  |
| 17 | Amoxicillin/pivsulbactam | |  |  |
| 18 | Amoxicillin/sulbactam | |  |  |
| 19 | Ampicillin/bacillus coagulans/cloxacillin | |  |  |
| 20 | Ampicillin/cloxacillin | |  |  |
| 21 | Ampicillin/cloxacillin/lactobacillus acidophilus | |  |  |
| 22 | Ampicillin/cloxacillin/saccharomyces boulardii | |  |  |
| 23 | Ampicillin/dicloxacillin | |  |  |
| 24 | Ampicillin/dicloxacillin/lactobacillus acidophilus | |  |  |
| 25 | Ampicillin/flucloxacillin | |  |  |
| 26 | Ampicillin/lidocaine/sulbactam | |  |  |
| 27 | Ampicillin/oxacillin | |  |  |
| 28 | Ampicillin/sultamicillin | |  |  |
| 29 | Ascorbic acid/metamizole sodium/penicillin g /streptomycin | |  |  |
| 30 | Azithromycin/cefixime | |  |  |
| 31 | Azithromycin/cefixime/lactobacillus acidophilus | |  |  |
| 32 | Azithromycin/cefpodoxime proxetil | |  |  |
| 33 | Azithromycin/fluconazole/secnidazole | |  |  |
| 34 | **Azithromycin/levofloxacin** | |  |  |
| 35 | **Azithromycin/ofloxacin** | |  |  |
| 36 | **Benzyl penicillin/streptomycin** | |  |  |
| 37 | **Bromelains/doxycycline/lactobacillus reuteri/lactobacillus rhamnosus/ornidazole** | |  |  |
| 38 | Bromhexine/sulfamethoxazole/trimethoprim | |  |  |
| 39 | Cefaclor/clavulanic acid | |  |  |
| 40 | Cefadroxil/clavulanic acid | |  |  |
| 41 | Cefadroxil/trimethoprim | |  |  |
| 42 | Cefalexin/trimethoprim | |  |  |
| 43 | Cefdinir/clavulanic acid | |  |  |
| 44 | Cefepime/sulbactam | |  |  |
| 45 | Cefepime/tazobactam | |  |  |
| 46 | Cefixime/cefpodoxime proxetil | |  |  |
| 47 | Cefixime/clavulanic acid | |  |  |
| 48 | Cefixime/clavulanic acid/lactobacillus acidophilus | |  |  |
| 49 | Cefixime/cloxacillin | |  |  |
| 50 | Cefixime/cloxacillin/lactobacillus acidophilus | |  |  |
| 51 | Cefixime/dicloxacillin | |  |  |
| 52 | Cefixime/lactobacillus acidophilus/ofloxacin | |  |  |
| 53 | Cefixime/levofloxacin | |  |  |
| 54 | Cefixime/linezolid | |  |  |
| 55 | Cefixime/moxifloxacin | |  |  |
| 56 | Cefixime/ofloxacin | |  |  |
| 57 | Cefixime/ornidazole | |  |  |
| 58 | Cefoperazone/sulbactam | |  |  |
| 59 | Cefoperazone/tazobactam | |  |  |
| 60 | Cefotaxime/sulbactam | |  |  |
| 61 | Cefpodoxime proxetil/clavulanic acid | |  |  |
| 62 | Cefpodoxime proxetil/cloxacillin/lactobacillus acidophilus | |  |  |
| 63 | Cefpodoxime proxetil/dicloxacillin | |  |  |
| 64 | Cefpodoxime proxetil/dicloxacillin/lactobacillus acidophilus | |  |  |
| 65 | Cefpodoxime proxetil/levofloxacin | |  |  |
| 66 | Cefpodoxime proxetil/ofloxacin | |  |  |
| 67 | Cefpodoxime proxetil/sulbactam | |  |  |
| 68 | Ceftazidime/sulbactam | |  |  |
| 69 | Ceftazidime/tazobactam | |  |  |
| 70 | Ceftazidime/tobramicin | |  |  |
| 71 | Ceftibuten/clavulanic acid | |  |  |
| 73 | Ceftriaxone/sulbactam | |  |  |
| 74 | Ceftriaxone/tazobactam | |  |  |
| 75 | Ceftriaxone/vancomycin | |  |  |
| 76 | Cefuroxime axetil/clavulanic acid | |  |  |
| 77 | Cefuroxime axetil/linezolid | |  |  |
| 78 | Cefuroxime axetil/sulbactam | |  |  |
| 79 | Cefuroxime/clavulanic acid | |  |  |
| 80 | Cefuroxime/sulbactam | |  |  |
| 81 | Chloramphenicol/tetracycline | |  |  |
| 82 | Ciprofloxacin/metronidazole | |  |  |
| 83 | Ciprofloxacin/ornidazole | |  |  |
| 84 | Ciprofloxacin/tinidazole | |  |  |
| 85 | Doxycycline/tinidazole | |  |  |
| 86 | Erythromycin/sulfamethoxazole/trimethoprim | |  |  |
| 87 | Erythromycin/trimethoprim | |  |  |
| 88 | Fosfomycin/trimethoprim | |  |  |
| 89 | Gatifloxacin/ornidazole | |  |  |
| 90 | Kanamycin/penicillin g | |  |  |
| 91 | Levofloxacin/metronidazole | |  |  |
| 92 | Levofloxacin/ornidazole | |  |  |
| 93 | Meropenem/sodium/sulbactam | |  |  |
| 94 | Meropenem/sulbactam | |  |  |
| 95 | Metronidazole/norfloxacin | |  |  |
| 96 | Metronidazole/spiramycin | |  |  |
| 97 | Metronidazole/tetracycline | |  |  |
| 98 | Mezlocillin/sulbactam | |  |  |
| 99 | Ofloxacin/ornidazole | |  |  |
| 100 | Oleandomycin/tetracycline | |  |  |
| 101 | Piperacillin/sulbactam | |  |  |
| 102 | Rifampicin/trimethoprim | |  |  |
| 103 | Sulfadiazine/sulfamethoxazole/trimethoprim | |  |  |

**Section-2: Demographics of the participants**

| Respondent’s Age: …………..Years | Gender: M/F |
| --- | --- |
| Working Experience: | < 1 Year  1-5 Years  >5Years |
| Qualification of dispenser: | 🞏 Diploma in pharmacy  🞏 Bachelor in pharmacy  🞏 Masters in pharmacy  🞏 Community Medical Assistant (CMA)  🞏 Nurse  🞏 Others |
